# Association of Age and Surgical Technique with the Quality of Life of Male Patients Treated for Abdominal Aorta Aneurysms: A Cross-Sectional Study

**DOI:** 10.3390/ijerph19116580

**Published:** 2022-05-28

**Authors:** Silvestra Barrena-Blázquez, Manuel Díez-Alonso, Luis Felipe Riera del Moral, Salvador Sanchez-Coll, Melchor Alvarez-Mon, Miguel A. Ortega, Fernando Ruiz-Grande

**Affiliations:** 1 Department of General Surgery, Príncipe de Asturias Hospital, 28801 Alcalá de Henares, Spain; manuelmariano.diez@salud.madrid.org; 2 Department of Surgery, Medical and Social Sciences, Faculty of Medicine and Health Sciences, University of Alcalá, 28801 Alcalá de Henares, Spain; 3 Department of Vascular Surgery, Nuestra Señora del Rosario Hospital, 28834 Madrid, Spain; luis.riera@salud.madrid.org (L.F.R.d.M.); ssanchezc@telefonica.net (S.S.-C.); fruizgrande@hotmail.com (F.R.-G.); 4 Department of Medicine and Medical Specialities, Faculty of Medicine and Health Sciences, University of Alcalá, 28801 Alcalá de Henares, Spain; mademons@gmail.com (M.A.-M.); miguel.angel.ortega92@gmail.com (M.A.O.); 5 Ramón y Cajal Institute of Sanitary Research (IRYCIS), 28034 Madrid, Spain; 6 Immune System Diseases-Rheumatology and Internal Medicine Service, University Hospital Príncipe de Asturias, (CIBEREHD), 28806 Alcalá de Henares, Spain; 7 Department of Vascular Surgery, Príncesa Hospital, 28834 Madrid, Spain

**Keywords:** abdominal aortic aneurysm, open abdominal repair, EVAR, SF-36, age, health-related quality of life

## Abstract

OBJECTIVES. The achievement of a good health-related quality of life (HRQoL) has become one of the primary objectives of medical–surgical interventions. The objective of this study is to determine the HRQoL of patients who underwent abdominal aortic aneurysm (AAA) surgery and to analyse the influence of age on HRQoL. MATERIALS AND METHODS. This is an observational cross-sectional study with 151 male patients who underwent an operation for AAAs between January 2013 and December 2020 in two hospital centres. HRQoL was assessed with the Spanish version of the 36-Item Short Form Survey (SF-36), starting in the month following the surgical intervention. Statistical analyses were performed using hypothesis tests and multivariate linear regression. RESULTS: The mean age of the patients was 73 years (SD: 7), and the mean interval between surgery and the interview was 37 months (SD: 27). The scores of the Physical Function (*p* = 0.001), Vitality (*p* = 0.016), Social Function (*p* = 0.014), and Mental Health (*p* = 0.007) dimensions of the SF-36 were significantly lower in the older age groups. In addition, the scores on the Physical Summary Component (*p* = 0.003) and the Mental Summary Component (*p* = 0.026) were significantly lower among individuals older than 70 years of age. The HRQoL in patients who underwent operations for AAAs was similar to that reported in the general population of Spain. Patients with an aorto-aortic shunt had better scores on the Physical Function (Beta: 10; *p* = 0.014) and Mental Health (Beta: 8.12; *p* = 0.040) dimensions than those who had an aorto-bi-iliac or bifemoral shunt, regardless of the age of the patients at operation. CONCLUSION: Among patients who underwent an operation for an AAA, there was a negative association between the age at operation and scores on the Physical Function, Vitality, Social Function, and Mental Health dimensions of the SF-36. The type of surgical technique influences the evolution of Physical Function and Mental Health scores, regardless of age.

## 1. Introduction

Abdominal aortic aneurysms (AAAs) are an important health problem due to their high frequency and severity. The prevalence of AAAs is 1.3% among men between 45–54 years of age and 12.5% among men between 75–84 years of age. The male-to-female ratio of AAAs is 4:1 [1]. The prevalence is positively associated with life expectancy and negatively associated with cardiovascular mortality. The most serious complication of an AAA is rupture, as it usually requires urgent surgical intervention and is associated with high mortality [2].

The treatment options and medical–surgical outcomes for AAAs are well known: medical treatment of cardiovascular comorbidities, open surgery with replacement by a prosthesis (OR), or implantation of an endoprosthesis (EVAR) [3]. The type of treatment is selected based on the size and morphology of the aneurysm, age, and comorbidities of the patient. OR has a morbidity and mortality ranging from 3–5% [2,4]. The initial morbidity and mortality of EVAR is lower (1–1.5%) and is usually indicated in patients with high surgical risk [5,6].

In recent years, it has been recognised that the objectives considered important for surgeons (morbidity, mortality, and long-term complications) can substantially differ, based on the patient’s perceived state of health after treatment [7,8]. Health-related quality of life (HRQoL) has become a prominent outcome measure and an aid for guiding patient expectations and decision making [7,8]. The 36-Item Short Form Survey Questionnaire (SF-36) is the most commonly used test to evaluate HRQoL in different arms of Medicine. It is generic, non-specific, and quantitative. A considerable amount of information has been published about the HRQoL of patients undergoing AAAs. Various authors have found a drop in all the parameters measured by the SF-36 questionnaire after surgical treatment of AAAs [9,10]. This drop is transient and the values of SF-36 return to preoperative levels after 6–12 months [11,12]. It is also known that age is negatively associated with all HRQoL indicators. However, few studies have analysed the long-term results of the SF-36 questionnaire in AAAs. Reported data indicate a progressive decrease in all the parameters of the SF-36 questionnaire, particularly those that reflect physical activity, as the age of the patients increases [13,14,15,16]. In addition, some publications have reported that patients treated by EVAR show a more marked decrease than OR patients [11,13]. Additionally, to date, studies have focused on middle-aged patients, and there are very few data on elderly patients [15,16]. Other specific health-related HRQoL instruments described to be used in vascular processes, such as the VascuQoL-6 questionnaire, have been used in peripheral arterial diseases and there is limited experience of AAAs [17]. Therefore, it is not known to what extent the age of the patient and the type of associated surgical intervention can influence HRQoL after the intervention. The objective of this study is to determine the influence of age and type of surgery on the HRQoL of patients with AAAs.

## 2. Materials and Methods

### 2.1. Design and Study Participants

Observational Cross-Sectional study. This study included patients who consecutively underwent treatment for AAAs between January 2013 and December 2020 at the Prince of Asturias University Hospital in Alcalá de Henares and at the Nuestra Señora del Rosario Hospital in Madrid. Patients undergoing scheduled, urgent, and preferential surgery were included. Patients with thoracic or thoracic-abdominal aneurysms and patients with occlusive involvement of the aorto-iliac sector were excluded. A cross-sectional survey on HRQoL after AAA surgical repair was conducted on patients who fulfilled the inclusion criteria by presenting the SF-36 questionnaire on quality of life and functional status. The results obtained for our patients were compared with the reference values of the Spanish version of the SF-36 for the population of males over 60 years old reported in a previous publication. The study adhered to the STROBE guidelines for designing and reporting cross-sectional observational studies [18].

Patients were identified from the computerised database that contains information about all patients who have undergone operations in the Vascular Surgery Unit. The study was approved by the Ethics Committee of the Prince of Asturias Hospital. The patients agreed to participate and signed the informed consent document.

Surgery was recommended when the aneurysm measured >5 cm in transverse diameter in the thoraco-abdominal-pelvic CT image performed with contrast; if the AAA growth was 1.5 times the reference value of the aortic diameter, or if the AAA expanded >0.5 cm/year; when it produced symptoms; or when symptoms of rupture were present. The choice of surgical technique—OR (laparotomy and implantation of aorto-aortic or aorto-bifemoral/bi-iliac prosthesis, according to the morphology of the vascular tree) or EVAR (implantation by minimally invasive route of an endoprosthesis with aorto-bi-iliac bypass)—was based on the preference of the surgeon and patient choice, although, in principle, EVAR was preferred in patients older than 70 years or with high surgical risk.

The clinical and demographic data of each patient were recorded in a computerised database in Microsoft Excel 2019 (v.19) R (Redmond, Washington, DC, USA). Data were collected on comorbidities, cardiovascular risk factors, aneurysm morphology, type of intervention, perioperative morbidity, mortality, and length of hospital stay.

### 2.2. Questionnaire SF-36

To determine the HRQoL, the validated Spanish version of the SF-36 questionnaire was used [18]. All patients were contacted by telephone to briefly inform them about the study and invite them to participate. All patients agreed to participate; the majority of them went to the hospital where an interview was conducted with the principal investigator. The investigator explained the objective of the study in detail, and the participants signed the informed consent form. Patients who could not travel to the hospital completed the questionnaire over the phone. All interviews were conducted between December 2019 and December 2020. A single survey was performed on all patients, and the time elapsed since the intervention was recorded.

The SF-36 consisted of 36 items that assessed 8 dimensions HRQoL: Body Pain (two items), Mental Health (5 items), Vitality (four items), Social Function (two items), General Health (five items), Physical Function (10 items), Emotional Role (three items), and Physical Role (four items). The scores obtained for each of the items were coded, aggregated, and transformed on a scale from 0 (worst health status) to 100 (best health status) according to the scoring and interpretation manual of the questionnaire [11]. The scores on these 8 dimensions were grouped into two Summary Measures: Mental Summary Component (Vitality, Social Function, Emotional Role, and Mental Health) and Physical Summary Component (Physical Function, Physical Role, Body Pain, and General Health).

### 2.3. Study Variables

The variables of main interest were the 8 dimensions of the SF-36 questionnaire. As potential confounders and effect modifiers, we considered the age of the patients when surgery was performed and the surgical technique. Variables of interest were collected for all participants.

### 2.4. Statistical Analysis

For each aspect of health included in the SF-36 test, the mean, median, percentiles (25, 50, 75), standard deviation, and proportion of individuals with the maximum (ceiling effect) and minimum (floor effect) scores were calculated.

For data analysis, patients were categorised into five-year age groups based on their age at the time of the intervention. The relationship between the SF-36 scores and the age of the patients was analysed in two ways. First, the results for our patients were compared with the reference values of the Spanish version of the SF-36 in the general population over 60 years old, published by López-García et al. [19]. This publication was based on a group of 3949 non-institutionalised subjects representative of the Spanish general population aged 60 years and over. Subjects were selected proportionally from all regions of Spain. The results of the SF-36 questionnaire were categorised by gender and age. We used ordinary least-squares adjustment to determine whether the trend of the association between the scores of the SF-36 dimensions and age was linear. Secondly, a linear regression analysis was performed for each of the health scales of the questionnaire: quality of life was taken as a dependent variable, surgical technique was taken as the independent variable, and age was included in the model as a covariate. The type of intervention was coded with the value 0 if it was an aorto-aortic bypass, and with the value of 1 if it was the aorto-bi-iliac or bifemoral bypass.

The Kruskal–Wallis test was used to compare the results of the SF-36 dimensions between the different age groups. Statistical Package for the Social Sciences (SPSS) (v.23) (IBM, Armonk, New York, USA) was used for statistical analyses.

## 3. Results

During the study period, 183 patients underwent surgery for AAAs: six women (2.7%) and 178 (97.3%) men. Of these, 112 were operated on with OR, and 70 with EVAR. Women were excluded for this study due to the reduced number of cases and to maintain homogeneity. In addition, between the intervention and the completion of this study, 18 patients (16.5%) in the OR group and 9 (13%) in the EVAR group died. Therefore, The HRQoL questionnaire was administered to the 151 male patients who remained alive: 93 (61.6%) in the OR group and 58 (38.4%) in the EVAR group. The mean age was 73 ± 7 years (median: 72 years, range: 94–48 years) (Table 1). The time interval between surgery and the interview was 37 months (SD: 27). Table 1 shows the scores of the eight SF-36 dimensions according to the age of the patients. The results of the Physical Role (*p* = 0.611), Body Pain (*p* = 0.210), and General Health (*p* = 0.119) dimensions were similar across age groups. The scores of the Emotional Role dimension were lower among older age groups, although the difference did not reach statistical significance (*p* = 0.059). In contrast, the scores of the Physical Function (*p* = 0.001), Vitality (*p* = 0.016), Social Function (*p* = 0.014), and Mental Health (*p* = 0.007) dimensions were significantly lower among older age groups (*p* for the linear trend *p* < 0.05). The results of the Physical and Mental Summary Component scores are shown in Table 2. Physical and Mental Summary Component scores were significantly lower in those older than 70 years of age (*p* = 0.003 and *p* = 0.026, respectively).

Table A4 of Appendix A shows the reference values of the Spanish version of the SF-36 in the population of Spanish males over 60 years of age, published by López-García et al. [19]. A decrease in scores was observed to be associated with the increasing age of the individuals in all dimensions except Mental Health (*p* for linear trend <0.001). The decrease is more pronounced for the Physical Function and Physical Role dimensions. A marked increase in the standard deviation of the means can also be observed in the scores of the older age groups.

When comparing the results of the two series, we observed higher average scores on the Physical Function and Body Pain dimensions among patients older than 70 years of age undergoing AAAs than among the control population. These patients also had lower scores for the Emotional Role dimension. However, the scores of all the health scales in our patients fell within the range of scores defined by the mean ± standard deviation described for the control population.

Linear regression revealed that the type of surgical technique was significantly associated with scores on the Physical Function (Beta: 10; *p* = 0.014) and Mental Health (Beta: 8.12; *p* = 0.040) dimensions (Table 3). Patients who underwent aorto-aortic bypass had higher scores than those who underwent aorto-bi-iliac or bifemoral bypass, regardless of the individual’s age at operation.

**Figure 1:**
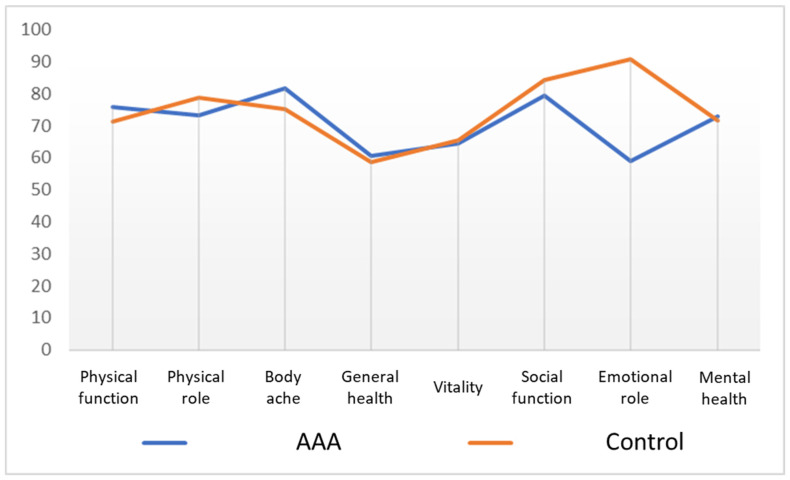
Mean scores in each of the dimensions of the SF-36 in patients operated on for abdominal aortic aneurysms (AAAs) and the general Spanish population (control).

Figure 1 shows the mean scores of each of the dimensions of the SF-36 in patients undergoing AAAs and in the Spanish population used as a control group.

## 4. Discussion

In recent years, attention has been devoted to the subjective perceptions of patients regarding their physical and psychological well-being after treatment [7,8]. For patients, their symptoms are the main concern, regardless of whether the condition is serious [16]. HRQoL assessments provide a basis for a holistic view of the patient and complement the traditional outcomes of morbidity and mortality. In this study, we used the Spanish version of the SF-36 to measure HRQoL [19,20]. This is the most widely used generic instrument in the international literature for measuring HRQoL.

The data obtained contain a minimal proportion of lost information, since the response rate was 100%. The overall mean scores are located in most of the dimensions of the SF-36 in the most positive extremes of the scoring range, but in no case in the maximum value, which indicates that the patients are not free of perceived health problems. The data show a progressive and statistically significant decrease in the scores on the Physical Function (*p* = 0.001), Vitality (*p* = 0.016), Social Function (*p* = 0.014), and Mental Health (*p* = 0.007) dimensions as the age at operation increases. In addition, the scores of the Physical Summary Component and Mental Summary Component scores were significantly lower in the age groups over 70 years.

These results suggest a loss of functional capacity associated with increasing age. A difference of 5 points on any scale is considered clinically and socially relevant [21]. In our series, we found that, for the Physical Function dimension, the difference between the oldest and youngest groups was 25.19 points; for the Vitality dimension, it was 20.58; and for the Social Function dimension, it was 15.39 points. For mental health, the difference was 14.56 points, which quantifies the deterioration of HRQoL.

We compared the results of our series with the reference values of the Spanish version of the SF-36 among adult males over 60 years of age, published by López-García et al. [19]. In this control population, an increase in age is associated with a decrease in scores for all dimensions, except Mental Health. The decrease is more pronounced in the Physical Function and Physical Role dimensions and reflects the loss of functional capacity that accompanies progressive ageing; the Mental Component scores were affected in a milder way.

The results obtained in our sample are comparable to those of the control population, even though the scores of some dimensions are higher in our series. We found better results for the Body Pain dimension, in all age groups, and for the Physical Function dimension among patients in the groups between 60 and 64 years and between 70 and 79 years. We also found better results for the General Health dimension among patients between 70 and 79 years old and for the Mental Health dimension among octogenarian patients. In contrast, patients of all ages showed worse scores on the Emotional Role dimension, and patients in the groups between 60 and 64 years and 70 and 79 years showed worse scores for the Social Function dimension.

The observed differences cannot be attributed to the method of administering the questionnaire, as in the study by López-García et al. [19]. The interviews were conducted in person in the homes, and in our study, 90% of these were conducted in the hospital, with the remainder being conducted via telephone. A possible explanation for the observed differences can be found in the clinical elements of our study. It must be taken into account that our patients had gone through a serious life experience, which predictably led them to especially assess their situation after the surgical intervention. The scores of the questionnaire can reflect the benefits provided by the treatment. This fact has been previously verified for patients undergoing cardiac and cardiovascular surgery. In one study, the scores were compared before and after treatment, and it was found that patients showed higher scores after the intervention on three of the scales with the highest content of physical factors (physical function, physical role, and body pain) [22].

It is very interesting that not all the variation in the scores of the SF-36 dimensions was due to the effect of age, but that the surgical procedure also exerted a significant effect, especially in the Physical Function (Beta: 10; *p* = 0.014) and Mental Health (Beta: 8.12; *p* = 0.040) dimensions. Patients who underwent an aorto-aortic bypass tended to have higher scores than those who underwent an aorto-bi-iliac or bifemoral bypass, regardless of the age of the individuals. This finding is consistent with several previous studies that showed that, in the long term (more than one year after the intervention), patients who undergo surgery using the EVAR technique have a lower score on the Physical Function dimension and on the scales of the Summary Component, than patients who undergo OR [13,23,24,25]. The EVAR technique is usually indicated in patients with high surgical risk, but is associated with a high incidence of reoperations and long-term complications [5,6].

The SF-36 is the most commonly used tool to evaluate HRQoL. It is a generic test; it is not specific to a specific pathology and has been used in various processes. The validity of the results has been verified, and it is widely accepted. Its generic design allows an objective quantification of HRQoL that facilitates the description of results and the comparison with other series and the comparison with the values detected in control groups, representative of the general population of each country [25]. Some studies have questioned the usefulness of the SF-36 among older people, as older multimorbid vascular patients usually find it very hard answering all items of the SF-36. [26]. However, we noted a high degree of understanding of the questionnaire for the patients of our study and all participants completed all its items, which demonstrates that it is a useful instrument, especially when it is administered through a personal interview to a group of selected and motivated patients. It would be desirable to define a pragmatic, simple, and reliable way to evaluate patient-reported outcomes in standard care, as such a tool is missing in AAAs at this moment [27].

The main problem of the SF-36 is its relative complexity in processing the raw data provided by the patient. Therefore, simplified derivations of the test have been described, such as the SF-8, which seems to provide comparable results [28,29]. On the other hand, since it is a nonspecific test, it can lose information that could be captured by tests designed for specific pathologies. In the field of AAAs, specific tests have been described that allow us to obtain qualitative information on the degree of HRQoL [16,30]. However, their value has not been verified, and they also have a greater complexity of application and interpretation of results. Among the areas of content not included in the SF-36 is sexual dysfunction, which could have negatively affected the results in the Emotional Role scale of our group compared to the reference population.

The main limitation of this study is that no information on HRQoL was collected prior to surgery, and was only obtained at a specific time after surgery. This type of design is not capable of capturing the entire evolution of the patient. It would be very useful to perform a randomised study with longitudinal development in the collection of information.

## 5. Conclusions

In conclusion, patients who underwent an operation for AAAs show lower scores on the Physical Function, Vitality, Social Function, and Mental Health dimensions of HRQoL as the age at which they are operated on increases. The type of surgical technique performed has a significant effect on the Physical Function and Mental Health scores, independent of the effect of age.

## Figures and Tables

**Table 1:** Scores of the Health Scales of the SF-36 questionnaire according to age groups.

Age Groups		Physical Function	Physical Role	Body Pain	General Health	Vitality	Social Function	Emotional Role	Mental Health
60–64 (*n* = 24)	MEAN	86.04	75.00	85.83	62.29	74.79	82.81	61.11	79.17
	SD	12.50	36.11	19.09	20.79	19.64	24.11	12.69	19.94
65–69 (*n* = 22)	MEAN	80.68	84.09	84.09	64.77	69.55	86.93	63.64	79.27
	SD	21.50	29.42	26.12	16.65	16.54	22.65	9.808	21.96
70–74 (*n* = 37)	MEAN	79.05	75.00	77.30	61.22	62.97	83.78	62.16	75.24
	SD	20.16	36.79	20.36	19.05	23.13	22.60	11.55	23.92
75–79 (*n* = 35)	MEAN	76.14	74.29	82.86	62.71	63.00	79.64	56.19	70.06
	SD	22.75	38.10	25.61	18.60	22.10	27.30	17.66	20.38
>80 (*n* = 33)	MEAN	61.21	62.12	80.91	53.18	56.21	67.42	53.53	64.61
	SD	24.11	44.68	25.78	17.62	19.76	27.23	20.31	19.80
Total (*n* = 151)	MEAN	75.83	73.34	81.72	60.50	64.34	79.55	58.94	72.93
	SD	22.30	38.04	23.46	18.79	21.37	25.63	15.60	21.77
*p*-value		0.001	0.611	0.210	0.119	0.016	0.014	0.059	0.007

**Table 2:** Summary Measures according to the Age of Patients Operated on for AAAs.

Age Groups		Physical Health	Mental Health
60–64 (*n* = 24)	MEAN	38.1208	37.7604
	SD	7.81883	7.78768
65–69 (*n* = 22)	MEAN	39.4176	37.2102
	SD	8.30605	7.26060
70–74 (*n* = 37)	MEAN	37.5000	34.5912
	SD	10.01091	8.93576
75–79 (*n* = 35)	MEAN	35.7827	34.8286
	SD	9.98618	8.53244
>80 (*n* = 33)	MEAN	30.5366	31.8636
Total (*n* = 151)	SD	11.78615	7.32420
	MEAN	35.9582	34.9354
	SD	10.24146	8.24213
*p*-value. Kruskal–Wallis test		0.003	0.026

**Table 3:** Linear regression analysis of the relationship between age and surgical procedure with the score on the quality of life scales (SF-36) of patients operated on for abdominal aortic aneurysm.

	Beta	*p*-Value
Physical Health	146	0
Constant	10.1	0.014
Surgical Procedure		
Age	−0.99	0
Physical Role	125	0
Constant	4.66	0.544
Surgical Procedure		
Age	−0.09	0.081
Body Pain	86.5	0
Constant	4.66	0.839
Surgical Procedure		
Age	−0.72	0.791
General Health	81.6	0
Constant	6.13	0.105
Surgical Procedure		
Age	−0.069	0.134
Vitality	114	0
Constant	5.84	0.163
Surgical Procedure		
Age	–0.70	0.002
Social Function	127	0
Constant	9.06	0.074
Surgical Procedure		
Age	−0.67	0.014
Emotional Role	87.8	0
Constant	−0.91	0.771
Surgical Procedure		
Age	−0.39	0.023
Mental Health	114	0
Constant	8.12	0.04
Surgical Procedure		
Age	−0.58	0.012

## Data Availability

The data used to support the findings of the present study are available from the corresponding author upon request.

## Appendix A

**Table A1:** Characteristics of the operated patients and associated pathologies.

	OAR (*n* = 93)	EVAR (*n* = 58)
Mean Age (SD)	71 (7)	76 (7)
Diabetes	16 (17.4%)	7 (12%)
Intermittent claudication	3 (3.2%)	1 (1.7%)
Lerich’s syndrome	1 (1%)	0
Ischemic heart disease	11 (11.8%)	11 (15.9%)
ACVA	2 (2.1%)	5 (8.6%)
EPOC	9 (9.6%)	8 (13.7)
Chronic renal failure	16 (17%)	7 (12%)
Hyperuricemia	5 (5.3%)	0
Hypothyroidism	4 (4.6%)	2 (3.4%)
Prostate pathology	16 (17.2%)	7 (12%)
SIZE OF THE ANEURISM		
40–56 mm	41 (44%)	24 (41.4%)
57–73 mm	28 (30%)	33 (56%)
73–90 mm	10 (10.7%)	1 (1.7%)
>90 mm	14 (15%)	0
LOCATION		
Juxta-pararenal	15 (16%)	0
Infrarenals	78 (84%)	69 (100%)
TYPE OF DERIVATION		
Bi-iliac aorto	47 (50.5%)	69 (100%)
Aorto-aortic	31 (33.5%)	0
Bi-iliac-femoral aorto	8 (8.7%)	0
Aorto-bifemoral	7(7.2%)	0
HOSPITAL STAY days (average)	10 (7)	4.14 (4)

**Table A2:** Scores of the Health Scales of the SF-36 Questionnaire according to age groups.

Age Groups		Physical Function	Physical Role	Body Pain	General Health	Vitality	Social Function	Emotional Role	Mental Health
60–64 (*n* = 24)	MEAN	86.04	75.00	85.83	62.29	74.79	82.81	61.11	79.17
	SD	12.50	36.11	19.09	20.79	19.64	24.11	12.69	19.94
	RANK	50–100	0–100	40–100	30–100	45–100	25–100	33–67	36–100
	MEDIAN	92.30	98.44	98.25	72.18	90.55	100.00	66.67	80.00
65–69 (*n* = 22)	MEAN	80.68	84.09	84.09	64.77	69.55	86.93	63.64	79.27
	SD	21.50	29.42	26.12	16.65	16.54	22.65	9.808	21.96
	RANK	20–100	0–100	20–100	30–85	40–95	25–100	33–67	20–100
	MEDIAN	87.50	100.00	100.00	67.50	75.00	100.00	66.67	84.00
70–74 (*n* = 37)	MEAN	79.05	75.00	77.30	61.22	62.97	83.78	62.16	75.24
	SD	20.16	36.79	20.36	19.05	23.13	22.60	11.55	23.92
	RANK	0–100	37–100	0–100	0–100	37–100	20–100	33–67	36–100
	MEDIAN	80.00	100.00	80.00	65.00	65.00	87.50	66.67	80.00
75–79 (*n* = 35)	MEAN	76.14	74.29	82.86	62.71	63.00	79.64	56.19	70.06
	SD	22.75	38.10	25.61	18.60	22.10	27.30	17.66	20.38
	RANK	5–100	0–100	0–100	20–95	15–100	0–100	33–100	20–100
	MEDIAN	85.00	100.00	90.00	65.00	70.00	87.50	66.67	76.00
>80 (*n* = 33)	MEAN	61.21	62.12	80.91	53.18	56.21	67.42	53.53	64.61
	SD	24.11	44.68	25.78	17.62	19.76	27.23	20.31	19.80
	RANK	0–95	0–100	10–100	15–85	20–90	13–100	0–67	32–100
	MEDIAN	60.00	100.00	100.00	50.00	60.00	75.00	66.67	68.00
Total (*n* = 151)	MEAN	75.83	73.34	81.72	60.50	64.34	79.55	58.94	72.93
	SD	22.30	38.04	23.46	18.79	21.37	25.63	15.60	21.77
	RANK	0–100	0–100	0–100	15–100	5–100	0–100	0–100	20–100
	MEDIAN	80.00	100.00	90.00	65.00	65.00	87.50	66.67	80.00
*p*-value		0.001	0.611	0.210	0.119	0.016	0.014	0.059	0.007

**Table A3:** Summary Measures according to the Age of Patients Operated on for AAAs.

Age Groups		Physical Health	Mental Health
60–64 (*n* = 24)	MEAN	38.1208	37.7604
	SD	7.81883	7.78768
	MEDIAN	41.6146	40.1250
65–69 (*n* = 22)	MEAN	39.4176	37.2102
	SD	8.30605	7.26060
	MEDIAN	43.8021	38.6875
70–74 (*n* = 37)	MEAN	37.5000	34.5912
	SD	10.01091	8.93576
	MEDIAN	41.7708	37.0000
75–79 (*n* = 35)	MEAN	35.7827	34.8286
	SD	9.98618	8.53244
	MEDIAN	38.6458	36.3750
>80 (*n* = 33)	MEAN	30.5366	31.8636
Total (*n* = 151)	SD	11.78615	7.32420
	MEDIAN	36.4583	34.0000
	MEAN	35.9582	34.9354
	SD	10.24146	8.24213
	MEDIAN	39.7917	36.2500
*p*-value. Kruskal–Wallis test		0.003	0.026

**Table A4:** Scores of the SF-36 Health Questionnaire Scales by Age in the Control Population. Adapted from López-Garcia E (10).

Scales	Age (Years)					
	60–64 (*n* = 369)	65–69 (*n* = 458)	70–74 (*n* = 385)	75–79 (*n* = 254)	80–84 (*n* = 153)	85 (*n* = 102)
Physical Health						
P5	25	25	10	5	10	0
P25	70	70	60	50	30	30
P50	90	90	80	80	60	60
P75	95	95	90	90	85	85
Mean (SD)	78.9 (28.3)	79.4 (27.0)	73.3 (27.0)	68.5 (29.8)	57.0 (30.8)	54.2 (34.5)
Physical Role						
P5	0	0	0	0	0	0
P25	100	100	75	75	25	0
P50	100	100	100	100	100	100
P75	100	100	100	100	100	100
Mean (SD)	85.0 (36.7)	84.2 (39.1)	77.5 (41.5)	76.1 (41.9)	71.0 (43.6)	69.0 (50.5)
Body Pain						
P5	22	22	22	22	12	21
P25	61	62	61	61	42	51
P50	100	84	84	74	80	74
P75	100	100	100	100	100	100
Mean (SD)	78.8 (30.8)	77.7 (30.0)	75.3 (28.7)	72.4 (27.5)	71.2 (32.3)	70.8 (30.8)
General Health						
P5	25	25	20	20	25	15
P25	45	47	45	40	40	40
P50	65	65	60	57	55	57
P75	77	77	72	72	67	72
Mean (SD)	61.3 (24.5)	61.9 (24.3)	59.0 (22.1)	56.5 (21.8)	55.1 (21.5)	56.1 (26.1)
Vitality						
P5	25	30	25	25	20	10
P25	55	60	50	45	40	45
P50	70	75	70	65	60	60
P75	85	90	85	80	80	80
Mean (SD)	68.6 (25.2)	71.0 (25.5)	66.5 (23.4)	63.4 (24.9)	58.5 (25.9)	59.0 (29.5)
Social Function						
P5	37.5	37.5	25	25	12.5	0
P25	87.5	87.5	75	75	62.5	50
P50	100	100	100	100	100	87.5
P75	100	100	100	100	100	100
Emotional Role						
P5	0	0	0	0	0	0
P25	100	100	100	100	100	100
P50	100	100	100	100	100	100
P75	100	100	100	100	100	100
Mean (SD)	92.4 (27.9)	91.4 (29.8)	92.1 (25.9)	89.6 (28.2)	87.6 (31.8)	83.1 (39.7)
Mental Health						
P5	36	40	44	36	36	32
P25	64	64	64	60	64	64
P50	80	80	76	76	72	80
P75	92	92	88	92	88	96
Mean (SD)	75.8 (22.1)	77.1 (23.7)	74.8 (19.3)	73.3 (21.4)	72.8 (19.7)	76.3 (24.1)
